# Infliximab therapy of relapsing tracheal stenosis in a pediatric patient with granulomatosis with polyangiitis: a case report

**DOI:** 10.1186/s13256-022-03370-2

**Published:** 2022-04-01

**Authors:** Alexander Schnell, Renate Ruppel, Christina Tremel, Matthias Galiano, Maria-Elena Meßbacher, Tobias Krickau

**Affiliations:** 1 Pediatric Pneumology and Allergology, Department of Pediatrics and Adolescent Medicine, University Hospital Erlangen, Friedrich-Alexander-University, Erlangen-Nuremberg, Germany; 2 Pediatric Nephrology, Department of Pediatrics and Adolescent Medicine, University Hospital Erlangen, Friedrich-Alexander-University, Erlangen-Nuremberg, Germany; 3 Department of Otolaryngology, Head and Neck Surgery, University Hospital Erlangen, Friedrich-Alexander-University, Erlangen-Nuremberg, Germany; 4 Pediatric Rheumatology, Department of Pediatrics and Adolescent Medicine, University Hospital Erlangen, Friedrich-Alexander-University, Erlangen-Nuremberg, Germany

**Keywords:** Granulomatosis with polyangiitis, Tracheal stenosis, Children, TNF-α inhibitor

## Abstract

**Background:**

Granulomatosis with polyangiitis is a granulomatous, necrotizing small-vessel vasculitis affecting both children and adults. However, subglottic tracheal stenosis appears more frequently in the pediatric cohort. To date, granulomatosis with polyangiitis is often treated with steroids, cyclophosphamide, azathioprine, or rituximab, but tumor-necrosis-factor-α-antagonistic drugs are increasingly gaining significance in treatment of refractory cases.

**Case presentation:**

We report the case of a 15-year-old Caucasian male diagnosed with proteinase-3-positive granulomatosis with polyangiitis with acute shortness of breath. X-ray and magnet resonance imaging showed extensive subglottic narrowing. Forced expiratory volume in 1 s was reduced to 50% of age norm, with massively increased effective airway resistance. The patient initially responded very well to high-dose steroids and maintenance therapy with azathioprine. He was subsequently treated with four doses of rituximab, and levels of proteinase 3 antibodies normalized. After 6 months of clinical remission, the patient presented again with acute respiratory symptoms. Again, he was treated with high-dose steroids, but showed poor clinical response this time. Therefore, we decided to commence a tumor-necrosis-factor-α-antagonistic treatment with infliximab, under which our patient achieved clinical remission and normalization of lung function parameters.

**Conclusions:**

The use of tumor-necrosis-factor-α-antagonistic agents might be a promising alternative for the treatment of refractory tracheal stenosis in pediatric patients with granulomatosis with polyangiitis.

## Introduction

Granulomatosis with polyangiitis (GPA) is a granulomatous, necrotizing small-vessel vasculitis associated with the presence of antineutrophil cytoplasmic antibodies (cANCA) affecting both adults and children. However, within the pediatric cohort incidence rates are found to be approximately sevenfold higher than in adults (1.8 versus 12.8 cases per million per year) [1], with proper diagnosis presenting a challenge for pediatricians. The exact pathophysiology remains elusive, but GPA is thought to appear in a two-stage course. It usually begins as a granulomatous disease of the respiratory tract in response to an exogenous or endogenous antigen, mainly affecting the ear–nose–throat (ENT) tract in the form of sinusitis/rhinitis, septal perforation, oral or nasal ulcerations, and otitis [2, 3]. Moreover, subglottic stenosis as a primary symptom of GPA has been reported to appear in around 4–10% and tends to be more frequently diagnosed in children than in adults [3–5]. The disease can progress to the systemic stage, resulting in diffuse vasculitis of one or several organs, such as kidney or lung. Yet, there are no significant differences between the pediatric and the adult cohort with regard to the pattern of clinical manifestations. However, if untreated, GPA is associated with a high mortality rate of up to 90% [6, 7]. Recommended therapy for remission induction usually is high-dose steroids in combination with cyclophosphamide, although the latter is of inferior relevance in the pediatric cohort because of fertility concerns. Since the RAVE trial (“rituximab in ANCA-associated vasculitis”) in 2010, B-cell-depleting therapy with rituximab has been a considerable possibility and often used as first-line therapy in children. For remission maintenance, low-dose steroids in combination with azathioprine, methotrexate (MTX), or RTX can be used [8]. However, there are several reports where a tumor necrosis factor α (TNF-α)-antagonistic therapy, either stand-alone or in combination with RTX, was successfully used for treatment of patients with refractory GPA [9, 10].

## Case presentation

We report the case of a 15-year-old Caucasian male presenting with unclear fever, relapsing otitides, and facial and nasal ulcerations for the first time in March 2020. Nasal biopsies showed extensive necrotizing granulomatous inflammation. cANCA/PR3 antibodies were highly elevated (360 U/ml, upper reference limit 20 U/ml). Imaging [sonography and magnetic resonance imaging (MRI)] revealed splenic infarction due to arteritis lienalis. There were no signs of kidney, lung, joint, or central nervous system involvement. The patient met the 2017 American College of Rheumatology criteria (ACR) for GPA. He was treated with high-dose steroids (1 g/day over 3 days), followed by azathioprine (2 mg/kg/day) and low-dose steroids as maintenance treatment. Owing to the splenic infarction caused by vasculitis of the splenic artery, he also received prophylactic antibiotic (penicillin) and anticoagulatory treatment (salicylic acid). The patient initially responded very well to the immunosuppressive treatment, and levels of PR3 antibodies normalized until May 2020. Consequently, glucocorticoids, salicylic acid, and penicillin could be discontinued. However, after discontinuation of steroid treatment, we detected another increase of PR3 antibodies up to 128 U/ml in June 2020, but owing to the good clinical apparition no further action was taken and levels of autoantibodies tended to decrease spontaneously.

**Fig. 1:**
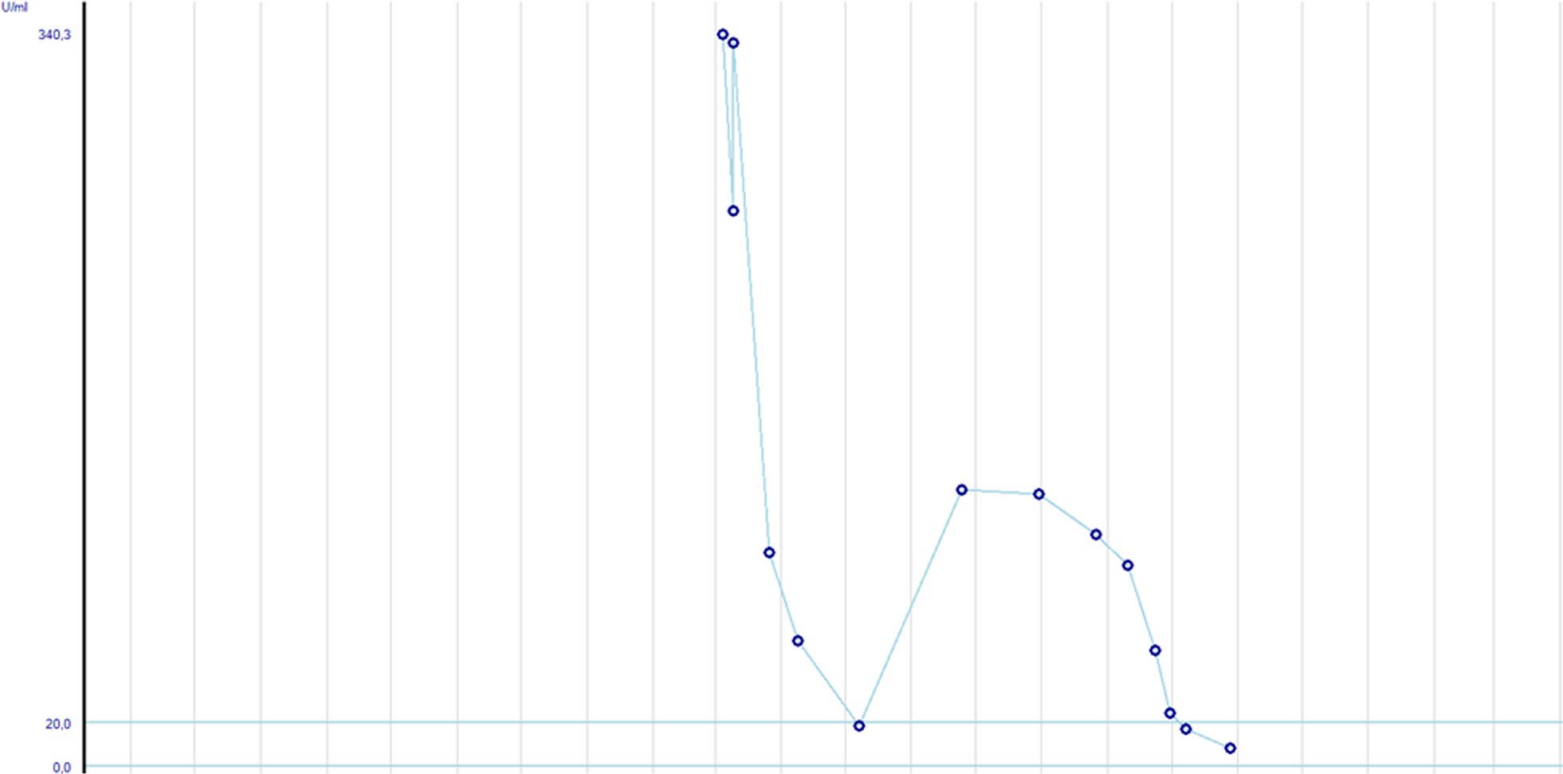
Levels of proteinase-3 antibodies over the past 15 months since initial presentation. After November 2020, no proteinase-3 antibodies were detectable. Blue lines indicate the normal range

In September 2020, he was admitted with a 2-week history of shortness of breath, even without physical activity, and inspiratory stridor. Moreover, he complained about hearing loss after discontinuation of glucocorticoid treatment. Lung function testing showed severe obstruction of the upper airways [forced expiratory volume in 1 s (FEV_1_) 50% of age norm] and a massively increased airway resistance [effective airway resistance (sR_eff_) 1018% of age norm]. MRI revealed a circular subglottic tracheal narrowing over a length of 2 cm. The levels of the beforehand-elevated PR3 antibodies showed no further increase (93 U/ml). We initiated a high-dose steroid treatment for 3 days followed by four subsequent doses of rituximab (RTX, 375 mg/m^2^, cumulative dose: 4 × 700 mg) in 4-week intervals for remission induction according to the therapy protocol of the RAVE study [11]. Clinical apparition and lung function parameters clearly improved under therapy, but the patient still reported shortness of breath under heavy physical activity and the flow profile in the body plethysmography still showed signs of tracheal stenosis. To match the decreasing dosages of oral glucocorticoids, he received inhalative corticosteroids (ICS; budesonide) together with a slow-acting bronchodilator (formoterol) in addition to the ongoing maintenance therapy with AZA on the occasion of a routine appointment in our pediatric pneumology department in November 2020. At that time, levels of PR3 antibodies also reached the normal range (Fig. 1).

**Fig. 2:**
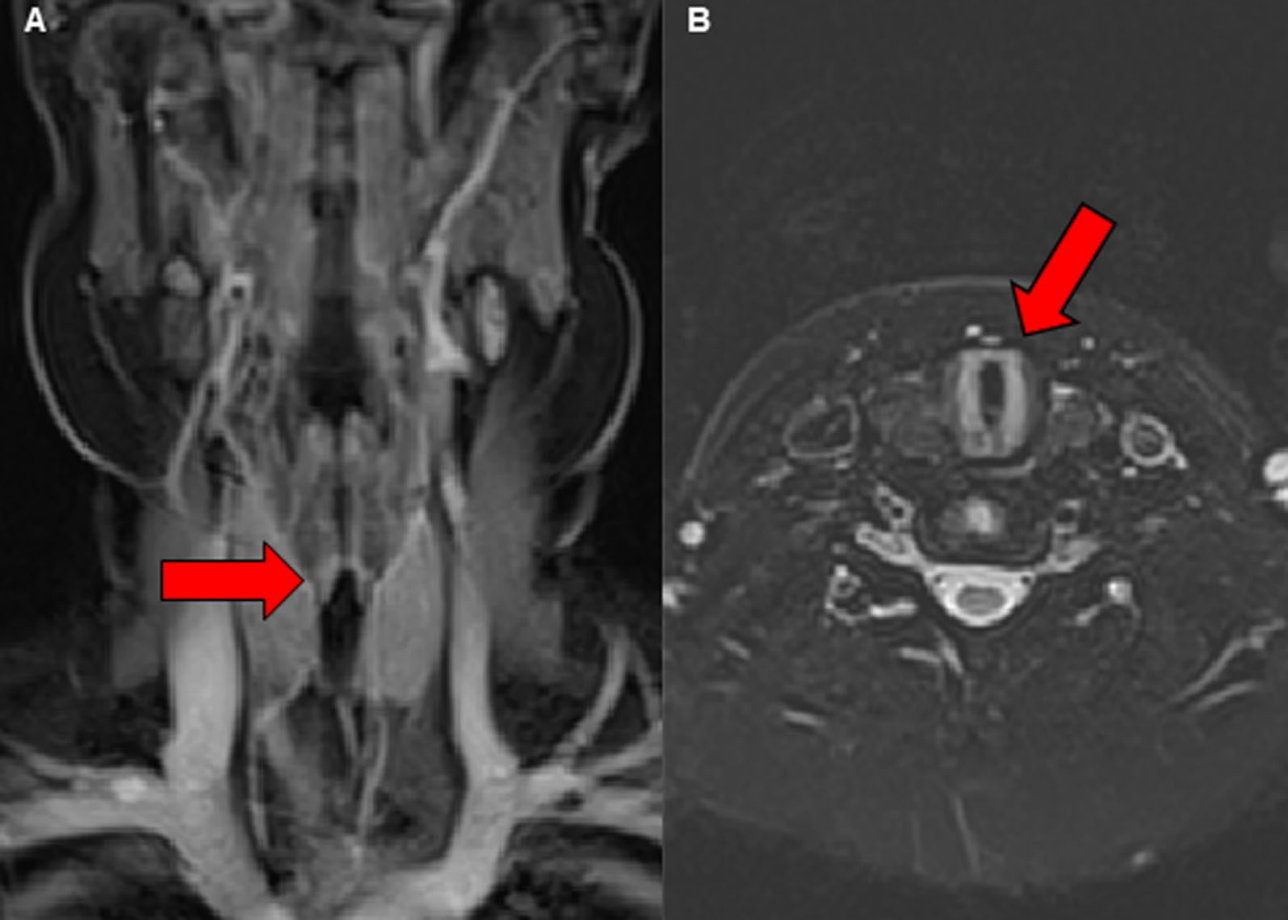
Radiological imaging of the subglottic tracheal stenosis in frontal (**A**) and transverse (**B**) plane. Abnormal findings are marked with red arrows

**Fig. 3:**
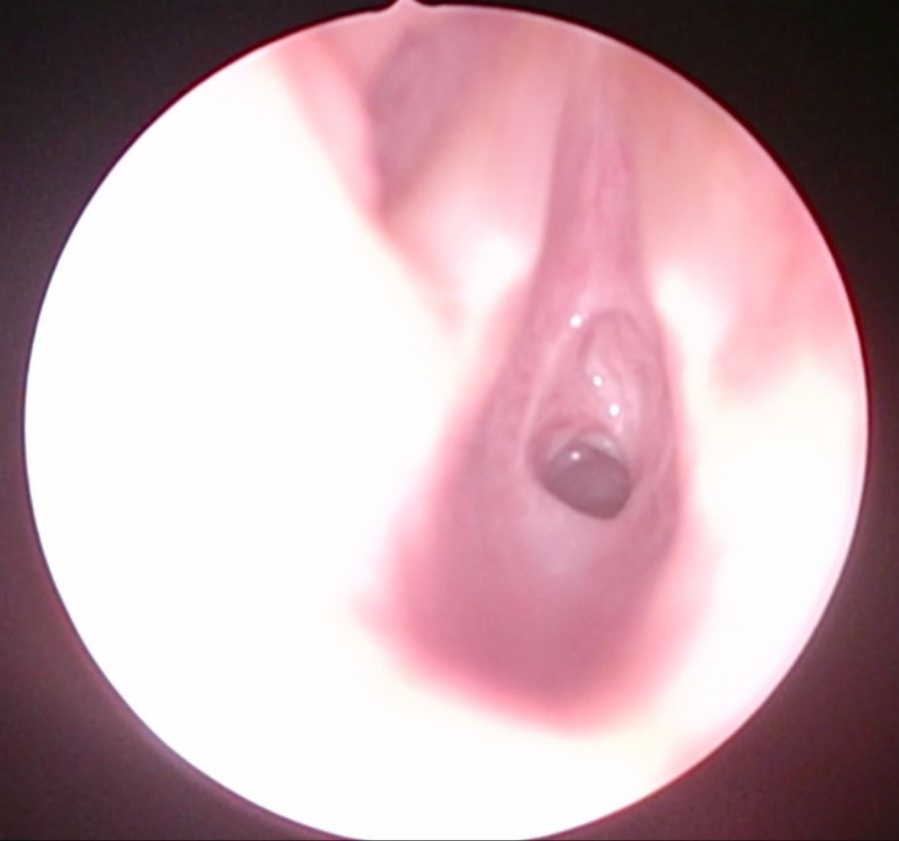
Fiberoptic imaging of the subglottic tracheal stenosis during laryngoscopy

**Fig. 4:**
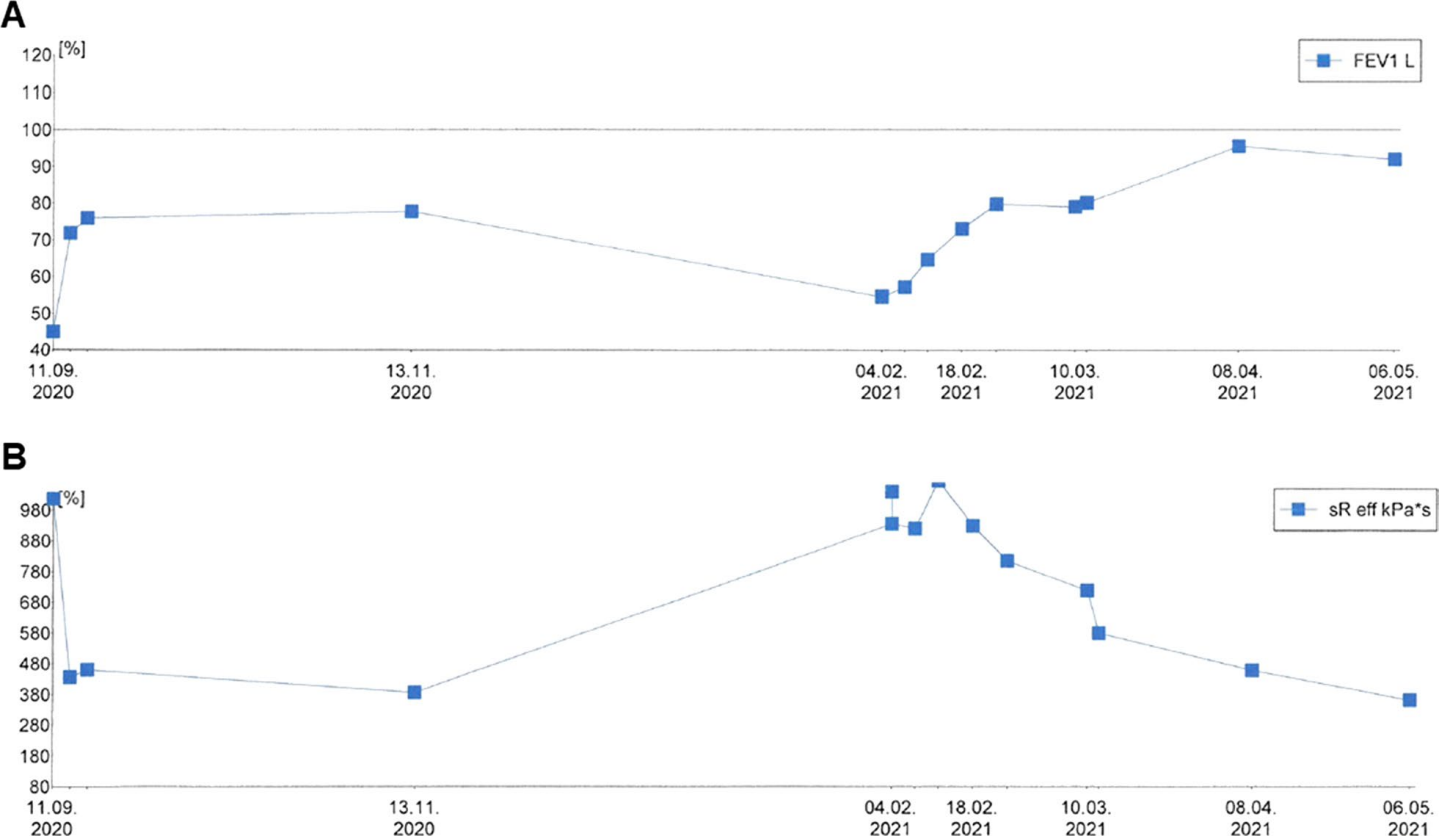
Development of the forced expiratory volume in 1 s (**A**) and effective airway resistance (**B**) over the past 12 months

After 3 months, in February 2021, his obstructive respiratory symptoms worsened as he complained about increasing inspiratory stridor. Blood gas analysis revealed a mild CO_2_ retention, and his lung function parameters showed a deterioration to similar values as in September 2020 (FEV_1_ 55% of age norm, sR_eff_ 1039% of age norm). Again, we performed an MRI, which showed a circular subglottic tracheal narrowing over a length of 1.5 cm (Fig. 2). To exclude further affection of the lower airways, we performed a chest computed tomography (CT) scan. Direct laryngoscopy showed a Cotton–Myer grade III subglottic stenosis, beginning 1 cm below the vocal cords with a length of 1.5 cm (Fig. 3). A biopsy taken from the subglottic tracheal lesion revealed ongoing neutrophilic inflammation. Again, high-dose steroids were initiated, but, in contrast to the prior admission in September 2020, the patient responded poorly. After consideration of the increased risk of infection due to the severe acute respiratory syndrome coronavirus 2 (SARS-CoV-2) pandemic and the persistently low B-cell count after the first RTX treatment, we decided against a second RTX cycle or other therapeutic alternatives (for example, cyclophosphamide) and initiated a TNF-α-antagonistic treatment with infliximab with a starting dose of 6 mg/kg. In induction phase, he received the first three doses in 2-week intervals, followed by 4-week intervals in maintenance phase. Moreover, AZA dosage was adapted to body weight. Our patient responded very well to the TNF-α-antagonistic treatment as his respiratory symptoms resolved completely and his lung function parameters normalized entirely (FEV_1_ 92% of age norm, sR_eff_ 361% of age norm; Fig. 4). At his last appointment in May 2021, he was in complete clinical remission.

## Conclusion

The development of biological drugs such as RTX or IFX has revolutionized the treatment of inflammatory disorders since they allow a therapy strategy tailored to the specific pathomechanism of the underlying disease. In the case of ANCA-associated vasculitis with its formation of PR3 antibodies by autoreactive B cells, a CD20-depleting therapy with RTX has consequently been established as a reliable option for induction and maintenance therapy alike. However, there is a notable number of patients with persistent autoantibody production after B-cell depletion since RTX treatment only disrupts the generation of plasmablasts but leaves the existing long-lived plasma cells untouched [12, 13]. Moreover, RTX has been described to have poor effectiveness in cases of predominant granulomatous disease activity (such as subglottic tracheal stenosis) in contrast to vasculitis-mediated glomerulonephritis [14, 15]. Taking a closer look at the pathogenesis of GPA, TNF-α can be identified as playing a pivotal role in the formation of granulomata in the localized stage. Effector memory T cells (T_EM_) produce large amounts of T_H1_ cytokines such as IFN-γ and TNF-α in response to antigens in the respiratory tract, thereby recruiting neutrophils to the site of inflammation [16, 17]. Moreover, there is evidence that the granulomata in the ENT tract might serve as tertiary lymphoid follicles, resulting in abundant formation of autoantibodies directed against neutrophilic proteinase 3 (PR3)/cANCA [18]. Consequently, regarding the central implications of TNF-α in GPA, it appears reasonable to consider a TNF-α-antagonistic treatment with, for example, IFX as a potential therapy alternative. Previous studies support the efficacy of IFX therapy in refractory cases [19, 20], and also the combination of TNF-α blockade and B-cell depletion has been used successfully [21, 22]. However, data concerning the use of infliximab in children diagnosed with GPA are scarce. However, the fact that subglottic stenosis represents a not uncommon symptom of pediatric GPA in connection with the severe constraints that are placed on the daily life of patients highlights the importance of effective therapy strategies, especially with regard to refractory cases. Our case report supports the potential of the additional use of infliximab in steroid and RTX refractory cases, yet there is a strong need for further evidence for the safe use of IFX in children.

## Data Availability

Not applicable.

## Abbreviations

ACR: American College of Rheumatology
AZA: Azathioprine
cANCA: Antineutrophil cytoplasmic antibodies
CYC: Cyclophosphamide
FEV1: Forced expiratory volume in 1 s
GPA: Granulomatosis with polyangiitis
ICS: Inhalative corticosteroids
IFX -: Infliximab
MRI: Magnetic resonance imaging
MTX: Methotrexate
PR3: Proteinase 3
RAVE: Rituximab in ANCA-associated vasculitis
RTX: Rituximab
sR_eff_: Effective airway resistance
T_EM_: Effector memory T cells
TNF-α-: Tumor necrosis factor α
